# Targeting autophagy to overcome drug resistance: further developments

**DOI:** 10.1186/s13045-020-01000-2

**Published:** 2020-11-25

**Authors:** Haocai Chang, Zhengzhi Zou

**Affiliations:** 1 MOE Key Laboratory of Laser Life Science and Institute of Laser Life Science, College of Biophotonics, South China Normal University, Guangzhou, 510631 China; 2 Guangdong Provincial Key Laboratory of Laser Life Science, College of Biophotonics, South China Normal University, Tianhe District, 55 Zhongshan Avenue West, Guangzhou, 510631 China

**Keywords:** Autophagy, Drug resistance, Metabolic stress, p53, MAPK, miRNA, Therapeutic antibody, Histone deacetylase

## Abstract

Inhibiting cell survival and inducing cell death are the main approaches of tumor therapy. Autophagy plays an important role on intracellular metabolic homeostasis by eliminating dysfunctional or unnecessary proteins and damaged or aged cellular organelles to recycle their constituent metabolites that enable the maintenance of cell survival and genetic stability and even promotes the drug resistance, which severely limits the efficacy of chemotherapeutic drugs. Currently, targeting autophagy has a seemingly contradictory effect to suppress and promote tumor survival, which makes the effect of targeting autophagy on drug resistance more confusing and fuzzier. In the review, we summarize the regulation of autophagy by emerging ways, the action of targeting autophagy on drug resistance and some of the new therapeutic approaches to treat tumor drug resistance by interfering with autophagy-related pathways. The full-scale understanding of the tumor-associated signaling pathways and physiological functions of autophagy will hopefully open new possibilities for the treatment of tumor drug resistance and the improvement in clinical outcomes.

## Introduction

Autophagy is a process of self-digestion with highly conservative attributes from yeast to mammals during evolution, which allows cells to sequester cytoplasmic components and fuse with lysosomes for degradation to maintain cellular biosynthesis and energy demand during nutrient deprivation or metabolic stress. In the mammalian cells, the three best-characterized pathways of autophagy are chaperone-mediated autophagy, microautophagy and macroautophagy. The degradation process of chaperone-mediated autophagy is selective to erase the cytoplasmic proteins relying on the recognition by the heat shock cognate 70 (HSC70) chaperone with the recognizable peptide sequence motif (KFERQ) [1]. Microautophagy directly sequesters the degradation of proteins which are translocated into the lumen in the form of lysosomal membrane invaginations [2]. Macroautophagy sequesters the cytoplasmic proteins or organelles by the expanding phagophore, leading to the formation of the double-membrane autophagosomes, which subsequently fuse with lysosomes [3]. Macroautophagy, referred to as autophagy, is closely related to the occurrence and drug resistance of tumor and chosen to be discussed in the review. Extensive studies have revealed that various signaling pathways regulate the phenomenon of autophagy. The PI3K/AKT/mTOR, LKB1/AMPK and Beclin1 complex are the core regulator of signaling pathways on autophagy. However, recent studies also have showed that p53-related pathways, mitogen-activated protein kinase (MAPK)-related pathways, metabolic stress-induced signaling, microRNA-triggered signaling and lncRNA-triggered signaling also participate in autophagy regulation, which make the role of autophagy on therapeutic drugs more unpredictable because induction of autophagy in response to tumor therapeutics has been considered as a double-edged sword with pro-death and pro-survival functions. Specifically, autophagy can clean up mutated cells, damaged organelles and genomic instability to inhibit tumorigenesis in the early stage of tumor formation [4, 5]. In established tumors, autophagy can be enhanced as a response to tumor cells against nutrient deprivation, energy deficits, hypoxia stress and chemotherapeutic drugs, finally they gradually induce acquired resistance [5]. Additionally, some tumor cell types with high basal autophagic flux may emerge intrinsic resistance to chemotherapeutic drugs and other targeted therapies. Conversely, persistent or excessive autophagy can induce autophagic cell death in tumor therapy [6, 7]. There would therefore seem to be a contradiction for the impact of autophagy on tumor. In brief, a further understanding of the mechanisms and functions on autophagy will delineate the multiple roles of autophagy as a novel target for both cancer prevention and cancer therapy.

## Mechanisms of autophagy

**Fig. 1:**
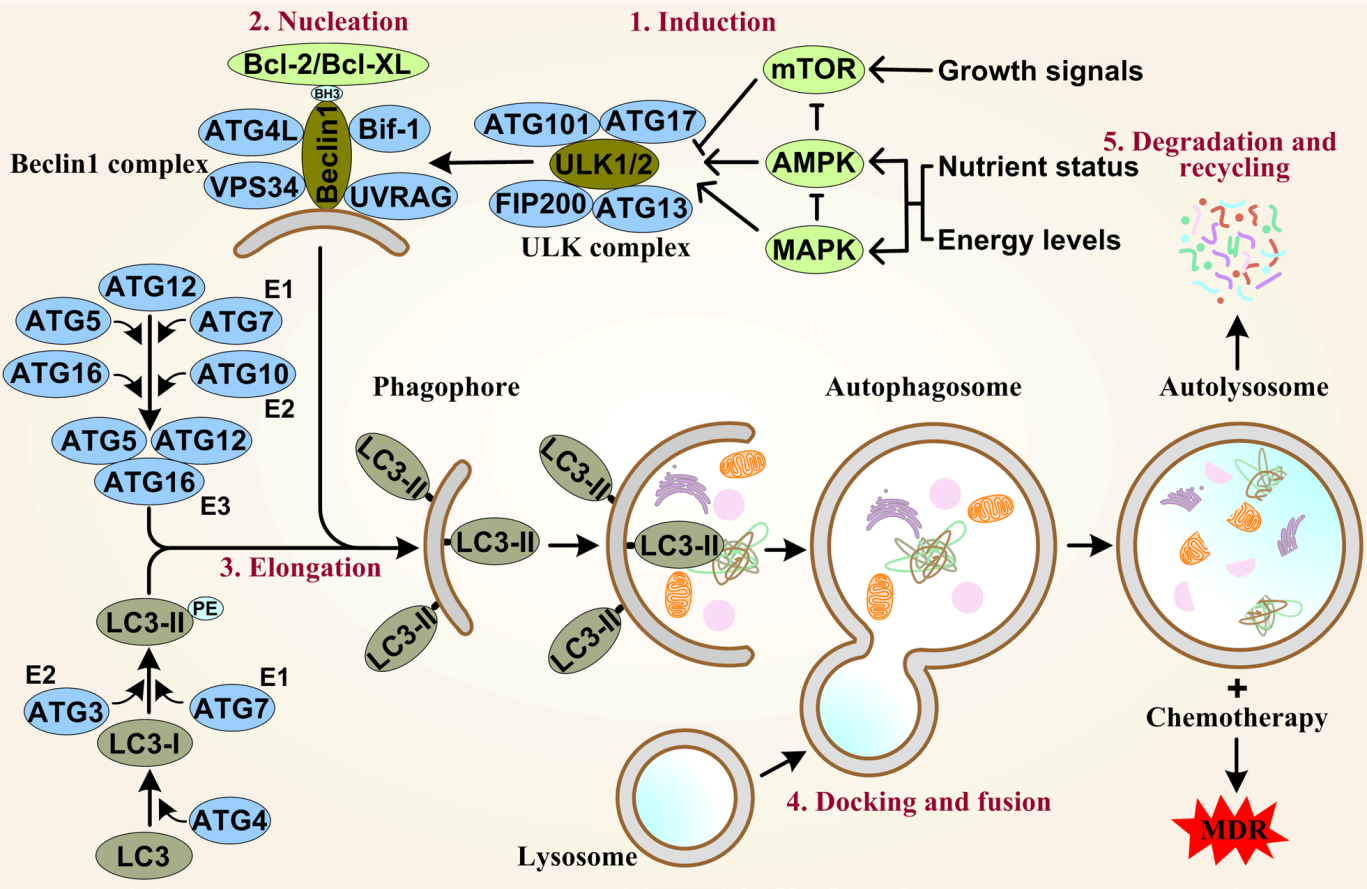
Overview of the autophagy-lysosomal degradation process. The initiation of autophagy is induced by a multiprotein complex consisting of ULK1/2, FIP200, ATG13, ATG17 and ATG101, which integrates stress signals from mTOR, AMPK and MAPK. The nucleation phase is controlled by Beclin1/VPS34/UVRAG/ATG4L/Bif-1 complex, which is negatively regulated by the antiapoptotic protein Bcl-2/Bcl-XL via its BH3 domain. The ATG12/ATG5/ATG16 and LC3-II are two dominate factors in the elongation phase to drive phagophore expansion. E3 ligase-like ATG12/ATG5/ATG16 multimeric complex is formed by the interaction of those proteins under the action of ligase E1 ATG7 and ligase E2 ATG10, and mediated membrane binding. LC3-II is from a cytosolic form of LC3-I by conjugating phosphatidylethanolamine (PE), which is catalyzed by ligase E1 ATG7 and ligase E2 ATG3, and then LC3-II is inserted into both outer and inner membranes of the growing phagophore. LC3-I is derived from pro-LC3 by ATG4 proteolytic cleavage to expose a C-terminal glycine and then conjugate PE. In the docking and fusion phase, autolysosome is generated by the fusion of autophagosome and lysosome. Finally the cargo-containing membrane and cytoplasm compartments are degraded and the essential biomolecules are recycled. Autophagy attenuates the damage caused by tumor therapeutic drugs and then produces multidrug resistance (MDR)

Autophagy in mammalian cells has been divided into five stages: induction, vesicle nucleation, vesicle elongation, fusion and degradation (Fig. 1). The initiating signals of autophagy to form the autophagosomes originate from the activated kinase Unc-51-like kinase-1/2 (ULK1/2, human homolog of yeast ATG1) [4, 8], and which then forms a pre-initiation complex with ATG13, ATG17, ATG101 and focal adhesion kinase (FAK) family interacting protein 200 (FIP200) under stress conditions [9]. ULK1/2 complex recruits the initiation complex composed of Beclin1 (human homolog of yeast ATG6), ATG14L, type III phosphatidylinositol (PtdIns) 3-kinase PIK3C3/vacuor protein sorting-34 (VPS34), autophagy and Beclin1 regulator-1 (AMBRA-1) and UV radiation resistance-associated gene protein (UVRAG), by phosphorylating Beclin1 to activate the autophagy-specific VPS34 [8]. Beclin1 and ATG14L synergistically facilitate the formation of autophagosomal membrane that is related to ATG5 and ATG12 [10]. Under the catalysis of E1-like enzyme ATG7 and E2-like enzyme ATG10, the formed ATG5/ATG12/ATG16(L) complex facilitates the recruitment and conversion of microtubule­associated protein light chain 3 (LC3-I) to the membrane-bound LC3-II form [11]. LC3-II also binds to the adaptor protein p62/sequestosome1 (SQSTM1) to degrade the ubiquitinated protein aggregates in autophagolysosome [12], and thus recycling amino acids and other metabolic building blocks to maintain cellular homeostasis.

### mTOR and Beclin1-related pathways

**Fig. 2:**
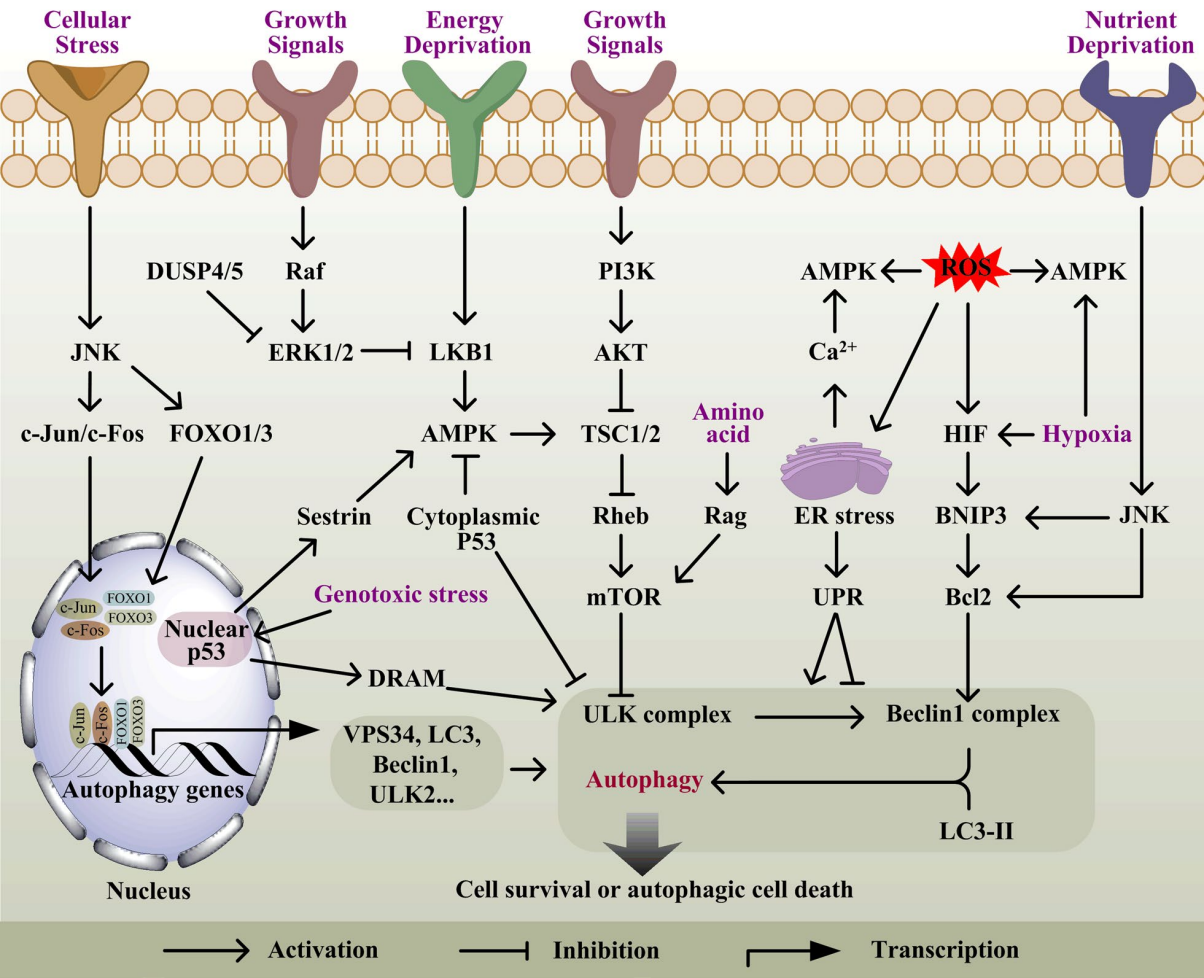
Regulation of autophagy. Multiple signaling pathways triggered by growth signals and energy status are integrated by mTOR, a core regulator of autophagic signaling, which initiate autophagy induction and inhibition. Cellular stresses, including genotoxic stress, ER stress, hypoxic stress and ROS, also elicit a series of intracellular and extracellular signal events and change the level of autophagic flux through distinct mechanisms

The mammalian target of rapamycin complex 1 (mTORC1) serves as the central modulator of autophagy. Activated mTORC1 inhibits autophagic cascade reaction by phosphorylating ATG13 and ULK1 to block the formation of pre-initiation complex [13], and by phosphorylating ATG14 to suppress the class III PI3K activity of ATG14-containing PIK3C3 [14]. mTORC1 integrates the upstream signaling of class I PI3K/AKT [15] and adenosine monophosphate kinase (AMPK) [16] (Fig. 2), which can sense the nutrient status and energy levels and deliver signaling to the key downstream 4EBP1 and p70S6K, which regulate protein translation. Activated AKT disrupts the accumulation of tuberous sclerosis protein 1/2 (TSC1/2) heterodimerization to inhibit the GTPase activity of Ras homolog enriched in brain (Rheb), which activates mTORC1 in its GTP-bound form [17]. Upon amino acids deprivation, the translocation of mTORC1 in Rag dependent manner to the lysosomal surface is restrained, which is a necessary event for its activation [18]. At low energy levels, the liver kinase B1 (LKB1, also known as STK11)/AMPK signal axis, as the metabolic sensor, activates TSC1/2 [19], then inactivates mTORC1 activity and ultimately increases autophagy. Also, the inhibition of mTORC1 by AMPK can trigger hypoxia-induced autophagy by hypoxia-inducible factor (HIF)-dependent and independent fashions [20, 21]. In addition, AMPK can also directly phosphorylate and activate ULK1 to organize the pre-initiation complex of autophagy [20, 22].

As an interactome scaffold, Beclin1 can self-associate to induce the formation of autophagosomes via its coiled-coil domain (CCD) [23, 24], or interact with antiapoptotic members of the Bcl-2 family to play an opposite role via Bcl-2-homology-3 domain (BH3) [25]. During starvation, the activated c-Jun N-terminal protein kinase 1 (JNK1) phosphorylates Bcl-2 to dissociate it from Beclin1 and activate autophagy [26]. Noteworthy, caspase activated by apoptotic signaling cleaves Beclin1 and PIK3C3 and then yields fragments which lack the capacity to induce autophagy [27, 28]. The fragments, in turn, enhance the apoptotic signaling to create an amplifying loop of apoptosis [28].

### p53-related pathways

Recent evidence has revealed that p53 plays a bidirectional role in regulating autophagy, which relies on its subcellular localization where nuclear p53 induces autophagy via its transcriptional activity while cytoplasmic p53 represses autophagy via its cytoplasmic functions. In the nucleus, p53 transcriptionally regulates 432 target genes, a host of which are autophagy genes [29]. Upstream of autophagy pathway, *AMPK β1*, *β2* and *γ* subunits, *PI3K p55γ* subunit, *TSC2*, phosphatase and tensin homolog (*PTEN*), DNA-damage-inducible transcript 4 (*DDIT4/REDD1)* and forkhead box O 3*a* (*FOXO3a)* are encoded by p53 target genes to regulate the AMPK and mTOR pathways [29, 30]. Within core machinery, numerous autophagy genes including *ULK1/2*, *ATG2b*, *ATG4a/c*, *ATG7*, *ATG10* and *UVRAG* are all bound by p53, which are directly involved in or regulate the composition of autophagic complexes [29]. Moreover, several lysosomal protein encoding genes, such as cathepsin D (*CTSD*), lysosomal-associated transmembrane protein 4A (*LAPTM4A*), tripeptidyl peptidase 1 (*TPP1*) and damage-regulated autophagy modulator (*DRAM*), are also as p53 target genes which may contribute to the generation of autolysosomes or the degradation of autolysosomal content [29, 31, 32]. Recently, several studies have shown that two other p53-family members, p63 and p73 also transcriptionally regulate the autophagy program. The phosphorylation of p63 induced by cisplatin transcriptionally upregulates the expression of *ULK1*, *ATG3*, *ATG5*, *Beclin1*, *ATG7* and *ATG10* and increases the control of autophagic pathways [33]. The activation of p73 induced by rapamycin starts up the expression of its target genes *TSC1*, *ATG5*, *PI3K p85α* subunit and insulin receptor (*INSR*) to induce autophagy [34, 35]. These comparisons among three p53 family members indicate that it’s a shared function for them to bind autophagy genes and promote the occurrence of autophagy.

Contrasting with the proautophagic functions of nuclear p53 by transcription-dependent mechanisms, cytoplasmic p53 restrains autophagy under various experimental conditions. Mutant p53 protein with a disrupted nuclear localization sequence, resulting in the cytoplasmic retention of p53, can efficiently inhibit autophagy [36]. Furthermore, in enucleated cells, deletion of p53 stimulates autophagy and cytoplasmic, not nuclear, p53 can manifest the capability to abolish the enhanced autophagy in p53^-/-^ cells. Under pro-apoptotic conditions, p53 rapidly translocates to the mitochondria and triggers mitochondrial membrane permeabilization (MMP) [37], most likely by engaging and repressing the antiapoptotic Bcl-2 family member Bcl-2/Bcl-XL or by activating its proapoptotic counterparts Bax/Bak, which leads to cytochrome *c* release [37, 38]. Accompanying p53 mutations on tumor progression, mutant p53 is detained in cytoplasm and loses its transactivation activity and the binding capability with Bcl-2 family proteins, instead, acquiring the autophagy-inhibitory action [36, 39], which leads to the tumor survival. Although the precise molecular mechanism by which cytoplasmic p53 inhibits autophagy has not been fully investigated, recent studies have showed that mutant p53 inhibits AMPK and activates mTOR, resulting in the suppression of autophagy [40, 41]. Moreover, mutant p53 can stimulate the stability of HIF-1, as an anti-autophagic protein, via intracellular reactive oxygen species (ROS)-mediated pathway [42]. Notably, mutant p53 cooperates with other transcription factors including E2F1, E2F4, SP1, NF-κB, NF-Y, and ZEB1 to promote expression of its target genes [43]. Cordani et al. showed that mutant p53 interacted with NF-κB p50 subunit, as a transcriptional repressor, and mutant p53/p50 complex was recruited onto the promoter of ATG12, an essential mediator of the formation of autophagosomal membrane, to inhibit autophagy [40]. Conversely, mutant p53 proteins are degraded by autophagy-dependent mechanism, instead of MDM2-dependent proteasomal degradation in physiological conditions [44], maybe resulting from the increased of mutant p53 stability [45].

### Mitogen-activated protein kinase (MAPK)-related pathways

Among mitogen-activated protein kinase (MAPK) family members, JNK and p38 MAPK (p38) are generally considered to induce cell growth arrest and apoptosis in response to the various extracellular stimuli, while extracellular signal-regulated kinase (ERK) activated by growth factors promotes cell proliferation and transformation [46]. JNK regulates autophagy through two distinct modes: on the one hand, the activation of JNK1, but not JNK2, phosphorylates Bcl-2 on multiple sites induced by starvation to dissociate it from Beclin1, which induces autophagy activation [26]. However, exposed to palmitic acid (PA) and hypoxic stress, it’s JNK2, not JNK1 promotes the induction of autophagy, most likely by its upstream protein kinase C μ (PKCμ) [47] and downstream adaptor protein p62 [48], labeling cytoplasmic cargo for autophagic degradation. Conversely, a recent study showed that targeted deletion of JNK1, JNK2 and JNK3 in neurons increased autophagy by a FOXO1/BNIP3/Beclin1 pathway, concomitantly increasing the expression of proapoptotic protein Bim [49]. On the other hand, the activated JNK can phosphorylate and then activate the transcription factor c-Jun/c-Fos, which transactivates the Beclin1 to induce autophagy [50]. Notably, as another key downstream transcription factors of JNK, FOXO transcribes multiple ATG genes to regulate autophagy. For instance, FOXO1 controls the transcription of VPS34 and ATG12, which involve in the autophagic initiation [51]. FOXO3 alters the transcription of many autophagy-related genes, including LC3, BNIP3, Beclin1, ULK2, ATG4b and ATG12L [52, 53].

In addition to inducing apoptosis, p38 MAPK also plays a dual role in the regulation of autophagy in response to chemotherapeutic agents. As a positive regulator, p38 MAPK signaling pathway regulates IFNγ-induced macrophage autophagy [54]. Under oxidative stress, the activity of p38α/β MAPK elicits the expression of ATG7 to regulate the autophagy-lysosome systems in muscle wasting [55]. As a negative regulator, phosphorylation of ATG5 at threonine 75 by the Gadd45β-MEKK4-p38 pathway inhibits starvation-induced autophagy [56]. Moreover, in senescent CD8^+^ T cells, p38 MAPK blockade induces an increase in autophagy to achieve the additional energy through enhanced interactions between p38 interacting protein (p38IP) and ATG9 [57]. As reported, some separate investigations have showed that aberrant ERK activation can promote autophagy in certain conditions. During starvation, ERK2 regulates nuclear localization and activity of TFEB, a master gene for lysosomal biogenesis, which significantly increases the number of autophagosomes [58]. Also, a recent study reported that ERK8 induces autophagy via interacting with LC3 and GABARAP [59]. Conversely, ERK1/2 inhibition activates the signaling axis LKB1/AMPK/ULK1 to stimulate autophagy in pancreatic ductal adenocarcinoma [60, 61]. As a speculation, the dual role of p38 MAPK and ERK pathway, depending on the cell types and stimulus, may control the balance between apoptosis and autophagy in response to genotoxic stress.

### Metabolic stress-induced signaling

**Fig. 3:**
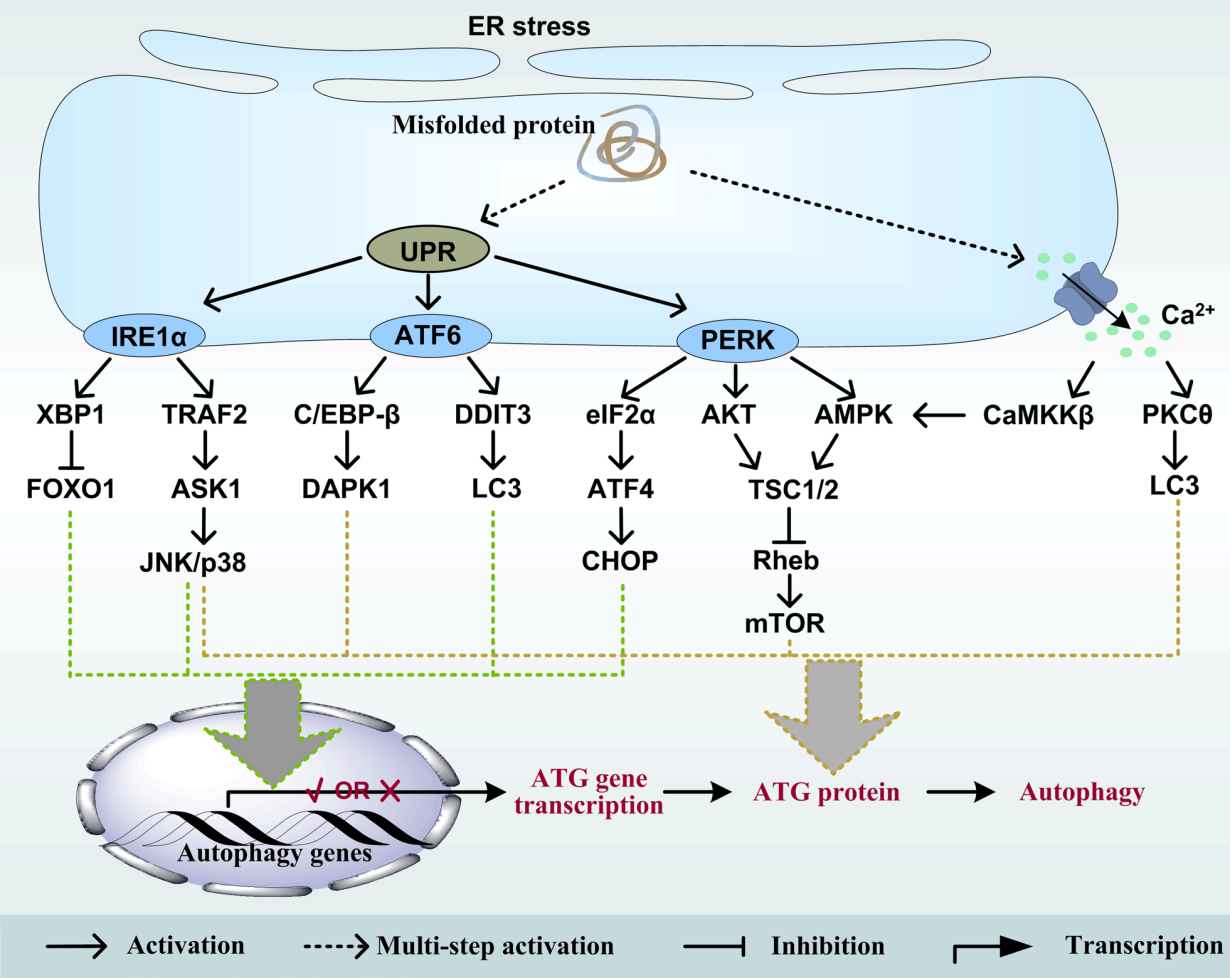
Regulation of autophagy by ER stress. Four major signaling pathways, including IRE1α, ATF6, PERK and Ca^2+^-related pathways implicated in ER stress-triggered autophagy. Among those pathways, UPR is triggered by the activation of the above three protein sensors (IRE1α, ATF6 and PERK) and regulates the transcription of autophagy genes and/or the function of autophagy protein. The release of Ca^2+^ from ER can activate autophagy via Ca^2+^/CaMKKβ/AMPK and PKCθ pathways. Dashed arrows indicate that the effect is multistep

The accumulation of unfolded proteins in endoplasmic reticulum (ER) causes ER stress and triggers the unfolded protein response (UPR), which orchestrates the recuperation of ER function. UPR are initiated by three protein sensors located on the membrane of ER, namely inositol-requiring enzyme 1α (IRE1α), activating transcription factor 6 (ATF6) and protein kinase R-like endoplasmic reticulum kinase (PERK), which are also in charge of the autophagy induced by ER stress (Fig. 3). IRE1α binds to TNF receptor-associated factor 2 (TRAF2), which then recruits and activates apoptosis signal-regulating kinase 1 (ASK1) [62]. Subsequently, ASK1 activates the JNK and p38 pathway during ER stress [63, 64]. Also, IRE1α-induced splicing of XBP1 negatively regulates the transcription factor FOXO1 by inhibiting its expression [65], and by interacting with FOXO1 to direct it toward proteasome-dependent degradation [66]. As a transcription factor, ATF6 interacted with transcription factor C/EBP-β is obligatory for death-associated protein kinase 1 (DAPK1) expression, which controls apoptosis and autophagy [67]. Other report has proved that DAPK1 modulates mATG9a trafficking during quinocetone treatment [68]. DNA-damage-inducible transcript 3 (DDIT3) activated by ATF6 upregulates LC3B expression by directly binding to its promotor region [69]. Moreover, the protein kinase PERK directly phosphorylates eIF2α and then specifically upregulated transcription factors ATF4 and its downstream target protein C/EBP-homologous protein (CHOP) to induce the expression of Beclin1, ATG5, ATG12 and the conversion of LC3B-I to LC3B-II [69–71]. The proapoptotic transcription factor CHOP acts the changeover switch between autophagy and apoptosis in the PERK pathway, because the inhibition of CHOP stimulates autophagy and suppresses apoptosis under ER stress [72]. Notably, the activation of PERK also stimulates the AKT- and AMPK-related signaling pathway to trigger autophagy [73, 74]. In addition to UPR, ER stress leads to the release of stored Ca^2+^ from ER to the cytosol in a free form, which could activate various protein kinases, as regulators of autophagy signaling. An increase in free cytosolic Ca^2+^ induced by Ca^2+^ mobilizing agents (ionomycin, ATP, thapsigargin and vitamin D compounds) activates Ca^2+^/calmodulin-dependent protein kinase kinase-β (CaMKKβ)-AMPK-TSC1/2-Rheb-mTORC1 signaling pathway and finally initiates autophagy [75]. Ca^2+^-dependent PKCθ activation is localized to LC3-containing dot structures and induces autophagy in response to ER stress [76].

Hypoxia, another metabolic stress, also induces autophagy in numerous normal and cancer cells under hypoxia (1% O_2_) and severe hypoxia (0.1% O_2_) conditions. In 1% hypoxia, autophagy is triggered through HIF-inducted BNIP3 and BNIP3L, which interact with Bcl-2 via their atypical BH3-domains [77, 78] (Fig. 2), and subsequently disrupt the association of Beclin1 and Bcl-2 [79]. In < 0.01% hypoxia, oxygen deprivation-induced autophagy in tumor cells is not dependent on HIF signaling and its target gene products BNIP3 and BNIP3L, but is supported by AMPK activity [80]. Alternatively, the UPR is activated by severe hypoxia to facilitate autophagy through regulating PERK/eIF2α/ATF4/CHOP pathway and then increasing transcription of the essential autophagy genes LC3 and ATG5 [81]. Thus, hypoxia signals can enter the energy metabolism and ER stress pathways to regulate autophagy, achieving maximum cell survival.

Accumulation of reactive oxygen species (ROS) and reactive nitrogen species (RNS) could cause oxidative stress, as an important inductor of autophagy, and there are characteristically high levels of ROS and RNS in cancer cells because of malfunction of the mitochondrial electron transport chain. Laura Poillet-Perez et al. have showed that ROS are essential for autophagy and directly oxidize the cysteine protease ATG4, which subsequently is inactivated to promote lipidation of ATG8 and autophagosome formation [82]. As reported, ROS specifically induce lysosomal Ca^2+^ release and then activate calcineurin-dependent TFEB-nuclear translocation, which regulates lysosome biogenesis and autophagy [83]. Notably, both high levels of ROS and RNS increase the activation of AMPK [84, 85] and MAPK [86, 87] to induce autophagy. However, the reduction of ROS/RNS accumulation will cause inhibition of autophagy [88]. ROS/RNS may specifically regulate these processes of pro-autophagy and anti-autophagy depending on their amount or cellular content.

### MicroRNA-triggered signaling

**Fig. 4:**
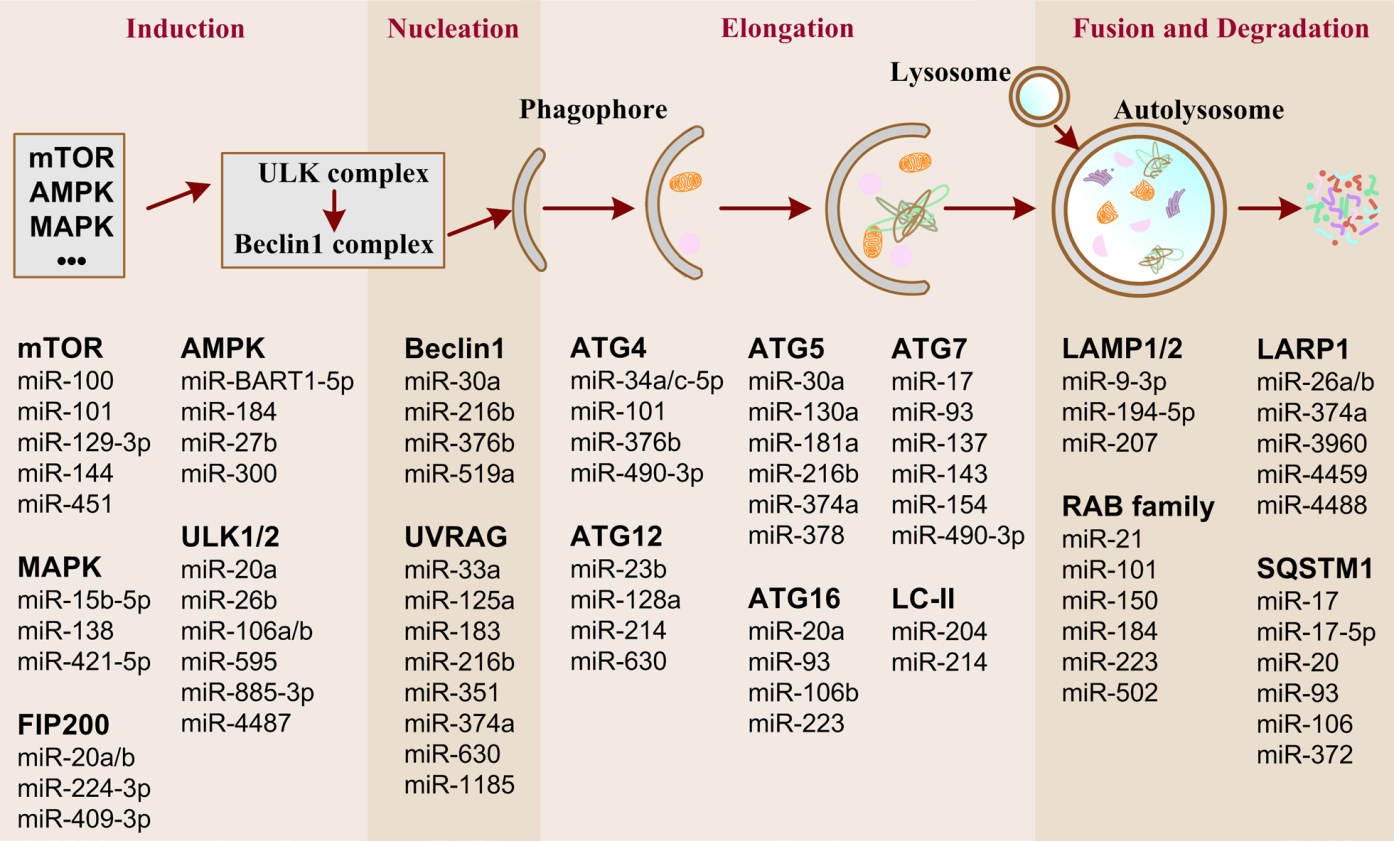
Overview of the miRNAs and specific targets involved in the regulation of autophagy at different stages

MicroRNAs (miRNAs) are small non-coding RNAs, which interact with targeted mRNAs mainly at their 3′ untranslated regions (UTR) and silence the gene expression in a post-transcriptional fashion. In the induction stage of autophagy, miRNA-106a and miRNA-885-3p exert their anti-autophagic function via targeting ULK1 [89], and ULK2 [90] respectively, and then impede autophagy induction (Fig. 4). In the second stage, various miRNAs, including miRNA-30a, miRNA-376b and miRNA-519a, target Beclin1 and reduce the autophagy activity of tumor cells induced by cisplatin [33, 91], rapamycin [92], and imatinib [93]. Moreover, miRNA-374a and miRNA-630 directly suppress autophagy by decreasing the expression of UVRAG, a component of the initiation complex [33]. In the third stage, miRNA-101 inhibits etoposide- and rapamycin-induced autophagy through targeting ATG4D [94], which inhibits the conversion of LC3-I to LC3-II. The protein levels of ATG5 are also dramatically reduced by miRNA-30a [93], miRNA181a and miRNA-374a [33] to block autophagic signaling. Another main LC3 homolog, LC3B, is directly regulated by miRNA-204 in clear cell renal cell carcinomas [95]. In the stage of fusion and degradation, targeted RAB5A by miRNA-101 to block autophagosome-lysosome fusion also inhibits etoposide- and rapamycin-induced autophagy [94]. miRNA-106 and miRNA-372 are identified as direct regulators of p62 [96, 97], which binds to LC3 to degrade the ubiquitinated protein aggregates in autophagolysosome.

miRNAs also target the mRNA of some genes in other signaling pathways. miRNA-146a increased by HIF-1α but not HIF-2α promotes hypoxia-induced autophagy through Bcl-2 [98]. miRNA-495-3p modulates the target gene GRP78 and its downstream mTOR to inhibit multidrug resistance in gastric cancer [99]. Moreover, the expression of DUSP4 and DUSP5, two negative regulators of MAPKs, is suppressed by miRNA-26a though directly interacting with their 3′-UTRs, which then boosts cytoprotective autophagy in the liver [100]. As upstream of mTOR and MAPKs, epidermal growth factor receptor (EGFR) expression is inhibited by miRNA-7 to induce autophagic cell death in human cancer cells [101]. p53-related signaling is also regulated by miRNA. For instance, miRNA-502 suppresses autophagy via a negative feedback regulatory loop of p53 in colon cancer [102].

### Long noncoding RNA-triggered signaling

Distinct from miRNA, long noncoding RNAs (lncRNAs) are longer than 200 nucleotides and their length with an advantage to bind target molecules at multiple sites in three-dimensional structures contributes to the complexity of signal network regulation composed of DNA, RNA and protein. Currently, accumulating evidence has indicated that lncRNAs participate in the regulating networks of autophagy by mediating the transcriptional and post-transcriptional regulation of autophagy-related genes. LncRNA growth arrest‑specific 5 (GAS5) activates autophagy to inhibit breast cancer proliferation, invasion and formation via upregulating ULK1/2 protein levels [103]. In uveal melanoma, tumorigenesis is inhibited by lncRNA ZNNT1-induced autophagy through upregulating ATG12 expression [104]. However, activated autophagy by lncRNA also promotes tumorigenesis and drug resistance. LncRNA nuclear-enriched abundant transcript 1 (NEAT1) activates autophagy via regulating ATG3 or Beclin1 expression and inhibits chemotherapeutic efficacy in hepatocellular carcinoma (HCC) [105] and colorectal cancer [106]. In breast cancer, activated autophagy by lncRNA H19, an imprinting lncRNA, contributes to tamoxifen resistance via upregulating Beclin1 expression [107]. One additional study indicated that lncRNA human plasmacytoma variant translocation 1 (PVT1) induces autophagy through increasing the protein and mRNA levels of Pygo2 and ATG14 and enhances cell resistance to gemcitabine in pancreatic cancer [108].

Conversely, some lncRNAs have exerted the effect of inhibiting autophagy to influence the occurrence and development of tumor. LncRNA cancer susceptibility candidate 9 (CASC9) suppresses autophagic cell death via the AKT/mTOR signaling and cell proliferation and tumor progression in oral squamous cell carcinoma [109]. LncRNA colon cancer-associated transcript-1 (CCAT1) is closely associated with a variety of cancers. A recent study has clearly shown that lncRNA CCTA1 induced autophagy inhibition for cell survival via increasing PI3K/AKT pathway in podocytes [110]. LINC00470, known as C18orf2, also activates AKT signaling, inhibits cell autophagy and promotes glioblastoma cell tumorigenesis and poor patient prognosis [111]. Beyond that, inhibited autophagy by lncRNAs could also limit tumor growth. In 42 tumor samples, lncRNA clarin 1 antisense RNA 1 (CLRN1-AS1) was downregulated compared to normal samples, and it abrogated prolactinoma cell proliferation by inhibiting autophagy in the way of Wnt/β-catenin signal deactivation [112]. Additionally, activated PI3K/AKT/mTOR signaling pathway and inactivated Beclin1 by lncRNA maternally expressed gene 3 (MEG3) inhibited autophagy and contributed to the cytotoxicity of adenosine in hepatoma HepG2 cells [113]. As discussed above, lncRNAs and autophagy have cross-regulation and dual effects on tumor because lncRNAs can increase or decrease autophagy, and the altered autophagy can further promote or inhibit tumor growth.

## Dual role of autophagy in tumor drug resistance

Autophagy plays a housekeeping role by recycling unnecessary proteins and damaged organelles in normal cells. In this regard, recycling by autophagy predictably provides a survival advantage to tumor cells during tumorigenesis. However, persistent and excessive autophagy could unexpectedly lead to caspase-independent autophagic cell death. Thus, autophagy induced by metabolic and therapeutic stresses, potentially inducing drug resistance, has a dual role as guarder and executioner with additional survival and death mechanisms.

### Autophagy as a guardian in tumor drug resistance

Extensive studies indicate that the upregulated autophagy not only enhances the survival of tumor, but also enhances the drug resistance of tumor in a wide range of tumor types. Although the mechanisms by which autophagy promotes tumor drug resistance are not fully understood, important clues are emerging. Apparently, autophagy induced by therapeutic agents supports tumor cell metabolism by recycling damaged proteins and organelles and then prevents DNA damage, as a result of inducing cancer drug resistance [114]. Recently, a direct evidence has been showed that the induction of autophagy-regulated DNA damage response via ataxia telangiectasia mutated (ATM)-mediated activation of DNA-dependent protein kinase catalytic subunit (DNA-PKcs) and poly(ADP-ribose) polymerase (PARP)-1 in response to capsaicin [115]. An enhanced DNA damage response is also induced by autophagy via homologous recombination (HR) repair pathway, which is a major way of repairing double-strand breaks [116]. Moreover, the stimulated autophagy induced by epirubicin (EPI) can increase drug efflux by P-glycoprotein (P-gp), encoded MDR genes which reduce intracellular concentrations of drugs, and downregulate the NF-κB signaling pathway, which decreases the rate of apoptosis, and thereby cause EPI resistance [117]. Another key protein encoded by MDR genes, multidrug resistance-associated protein 1 (MRP1) is also upregulated by ER stress-triggered autophagy for increasing intracellular drug efflux [118]. Aldehyde dehydrogenase 1A3 (ALDH1A3), as a detoxifying enzyme to drive acquired drug resistance, is also regulated by autophagy during temozolomide treatment [119]. As a prototypical damage-associated molecular pattern (DAMP) molecule, high-mobility group B1 (HMGB1) is released by the induced autophagy [120, 121], and promotes drug resistance in ovarian cancer [122], colorectal cancer [123] and lung cancer [124]. Thus, autophagy-mediated drug resistance is a multifactorial phenomenon involving cytoplasmic material renew, gene repair, alterations in drug concentration and metabolism and changes in the expression or activity of key protein.

Of particular importance, the development of drug resistance also involves the changes of apoptotic and survival signals. In tumor necrosis factor (TNF)-related apoptosis-inducing ligand (TRAIL)-resistant cells, the cytoprotective autophagy degrades a subunit of the active caspase-8 enzyme, an executive protein on mitochondrial apoptosis pathway [125]. p53, as a tumor suppressor, activates autophagy, whereas the activated autophagy in turn suppresses p53 [114]. As mostly reported, the induced autophagy is accompanied by the disruption of the Beclin1/Bcl-2 complex, and then Bcl-2 is released from the complex to enhance cell survival [126]. In this regard, the three types of molecular changes may also involve in autophagy promoting drug resistance: inactivation of proapoptotic factors, activation of antiapoptotic effectors and enhancement of survival signals. Namely, autophagy increases stress-induced damage threshold to buffer therapeutic stress and eliminates the signals of apoptosis, which both are required to induce cell death, thereby confers tumor resistance to apoptosis.

### Autophagy as an executioner in tumor drug resistance

Besides cell survival, autophagy can also induce cell death, namely autophagic cell death, caused by prolonged or sustained autophagy, which is defined as type II cell death preceded or accompanied by the large-scale autophagic vacuolization in cytoplasm and the resultant vacuolated appearance independent of apoptosis (type I cell death) and necrosis (type III cell death). Overexpression of autophagy genes may be a major factor in the death execution process by autophagy. For instance, enforced expression of Beclin1 induces excessive autophagy and promotes death of SW982 human synovial sarcoma cells [127]. The accumulation of p62/SQSTM1 induced by plant phytoalexin resveratrol triggers autophagic cell death in imatinib-resistant chronic myelogenous leukemia (CML) cells [6]. Additionally, the ATG5-ATG12 complex also accumulates to induce autophagic cell death in metformin-treated ovarian cancer cells [128]. Thus, autophagic cell death driven by autophagy genes is context-dependent and also induced in apoptosis-resistant cells, particularly in pro-apoptotic protein deficient cells. Moreover, autophagic cell death can be served as an alternative cell death approach when cells fail to undergo apoptosis, even in the drug resistance of tumor.

Signaling thresholds may be another crucial factor that dictates whether the autophagy trends the role of pro-survival or pro-death. The intensity of oncogenic Ras signaling is related to the outcome of senescence or autophagic cell death. Enforced expression of Ras upregulates the BH3-only protein Noxa as well as Beclin1 to promote autophagic cell death [129]. Overexpression prolidase induces massive autophagy that leads to cell death via upregulating ATG7, LC3A/B and Beclin1 [130]. Additionally, multiple signal pathways are activated at the same time to achieve the goal of excessive autophagy. Baicalein, a flavonoid with well-established anticancer properties, activates AMPK/ULK1 and downregulates mTORC1 complex components to induce autophagic cell death [7]. Notably, the high expression of caspase-10 and cFLIP_L_ in myeloma cells inhibits autophagic cell death by cleaving and inactivating BCLAF1, encoding Bcl2-associated transcription factor 1 [131]. The explanation for such discrepancies (pro-survival and pro-death using the same set of autophagy equipment) is a quantitative relationship in which upstream signaling molecules affect cellular responses by controlling the relative threshold of autophagy flux in response to external stimuli. Improved autophagy flux over the threshold (autophagic cell death) or decreased autophagy flux (blocking autophagy for survival) may be a novel strategy to treat tumor in autophagy-mediated drug resistance.

## Targeting autophagy against tumor drug resistance

**Fig. 5:**
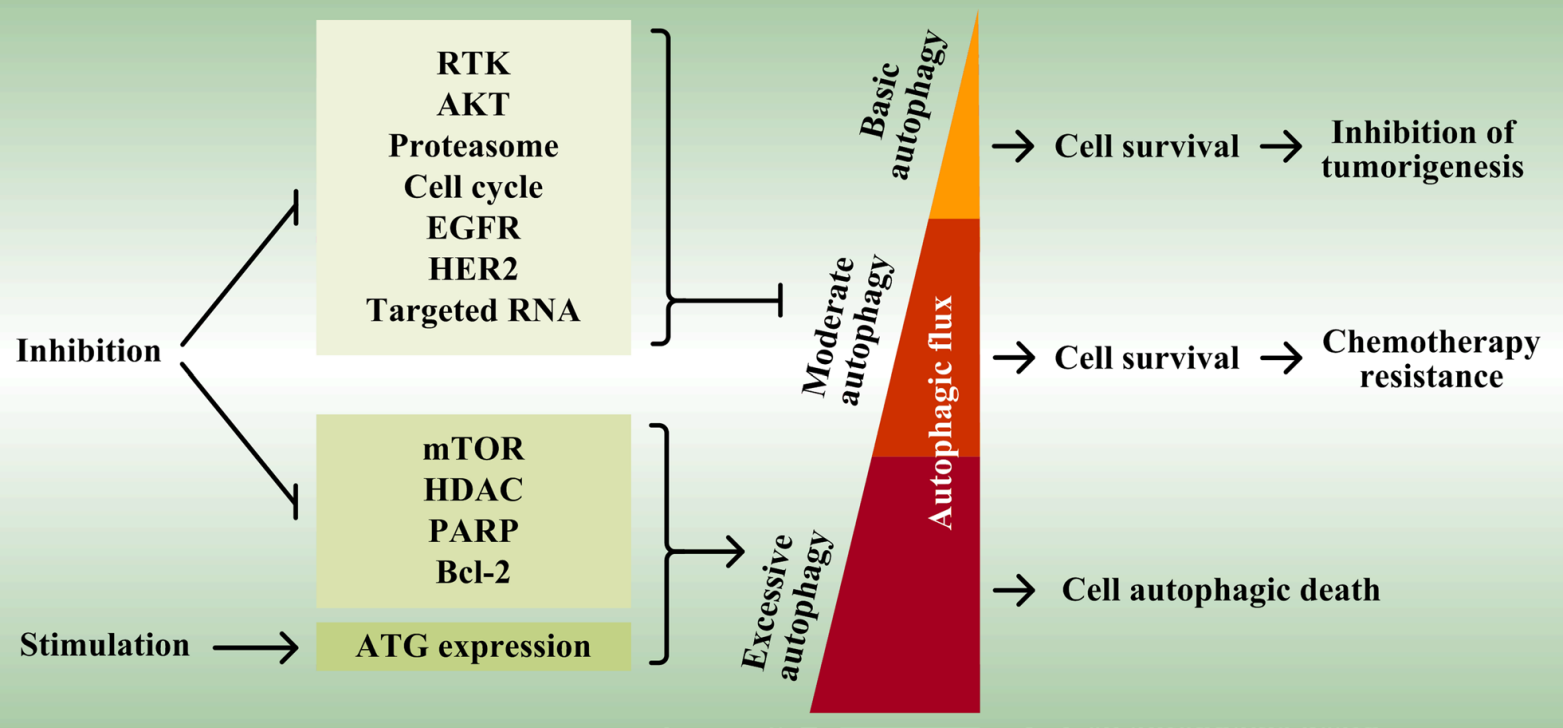
Employing autophagy against tumors. Basic autophagy recycles dysfunctional or unnecessary proteins and damaged or aged cellular organelles to provide constituent metabolites for cells, and inhibits tumorigenesis. The induction of moderate autophagy by drugs, which possess a higher autophagic flux and triggers drug resistant and even refractory cancer against the induced apoptosis, can be repressed by some chemical inhibitors or therapeutic antibodies (such as RTK inhibitors, AKT inhibitors, proteasome inhibitors, cell cycle inhibitors, miRNA, and EGFR and HER2 therapeutic antibodies). Conversely, other inhibitors (such as mTOR inhibitors, HDAC inhibitors, PARP inhibitors and Bcl-2 inhibitors) and enforced expression of ATG (such as ATG3, ATG4, ATG5, ATG9 and Beclin1) further improve autophagic flux and lead to cell autophagic death due to the excessive autophagy

Tumor therapeutic drugs primarily act by inducing apoptosis to kill cells. Based on the above description, basic autophagy protects cancer cells from chemotherapy, leading to drug resistant and even refractory cancer against the induced apoptosis. However, a high level of autophagy occurs for sustained or excessive metabolic cycle of intracellular components, which leads to excessive removal of essential proteins and cellular organelles, and finally causes caspase-independent autophagic cell death with benefit for the treatment of therapeutic drugs (Fig. 5).

### Autophagy inhibition facilitates the therapeutic efficiency

As therapeutic drug-promoted autophagy is supported by abundant substantial evidence, a new therapeutic strategy considering a combination with inhibiting autophagy has been proposed. Currently, studies to inhibit autophagy commonly employ the approaches of genetic silencing of ATGs and pharmacological inhibitors. Gene silencing using small interfering RNA (siRNA) usually knocks down Beclin1, ATG5, ATG7, ATG8 and ATG12 to inhibit autophagy and sensitizes the drug-resistant cancers [132–135], while pharmacological inhibitors generally use 3-methyladenine (3-MA), bafilomycin A1 (BafA) and chloroquine (CQ) to inhibit the formation of autophagosome [132–134, 136] and sensitize the resistant cancer cells to chemotherapy. 3-MA is an inhibitor of class III PI3K, VPS34, and thus it inhibits autophagy at an early stage. In contrast, BafA, a vacuolar H^+^-ATPase inhibitor, and CQ, a fusion inhibitor of autophagosome and lysosome, are in the late stage of autophagy to block cargo degradation.

Specific chemical inhibitors of receptor tyrosine kinase/Class I PI3K/AKT/mTOR pathway have been shown to be effective anti-tumor therapies. Imatinib and dasatinib, as tyrosine kinase inhibitors, are the standard treatments for CML, but a great many of patients don’t respond effectively. Pharmacologically blocking autophagy using CQ, or silencing of ATG5 and ATG7, could significantly enhance imatinib-resistance CML cell death [132, 136]. Dasatinib combined with autophagy inhibitors resulted in almost complete disappearance of phenotypical and functional CML stem cells [136]. As the dual Class I PI3K/mTOR inhibitor, NVP-BEZ235, synergized with autophagy inhibitors promotes apoptosis of drug-resistant tumors [133, 134]. Perifosine is an alkylphospholipid to inhibit AKT activity and exhibit antitumor activity. The combination of perifosine with CQ or NH_4_Cl enhanced apoptosis and the inhibition of tumor growth [137]. In clinical phase I trial, the combination of mTOR inhibitor temsirolimus and autophagy inhibitor hydroxychloroquine (HCQ), augmented cell death in patients with advanced solid tumors and melanoma [138]. The above discussion shows that the same effect can be achieved at any step in the suppression of receptor tyrosine kinase/Class I PI3K/AKT/mTOR pathway combined with autophagy inhibition. However, choosing which step in the pathway for combination therapy may need to consider tumor type and stage of tumor development.

Preclinical models showed that some proteasome inhibitors stimulated autophagy by the accumulation of misfolded proteins, namely ER stress. In agreement with the viewpoint, the inhibition of proteasome inhibitor (bortezomib and NPI-0052)-induced autophagy using 3-MA or CQ increased the levels of cell death in prostate cancer cell model [139]. Compared with wild-type animals, proteasome inhibitor epoxomicin exhibited an enhanced antitumor function in autophagy­defective Beclin1^+/–^ mice [135]. The next-generation proteasome inhibitors carfilzomib and oprozomib also induced autophagy and enhanced cell death when used with autophagy inhibitor CQ in head and neck squamous cell carcinoma tumor [140]. CQ stimulates a slow protein accumulation, localized to lysosomes, while bortezomib induces a rapid buildup of proteins where aggresome formation is in the cytosol [141]. The accumulation events often lead to mitochondrial disorder, accompanied by the release of cytochrome *c* and the activation of Apaf-1 containing apoptotic complex [142]. Thus, the combination of proteasome inhibition with autophagy inhibition can achieve stronger antitumor effect.

Cell cycle-mediated drug resistance is described that tumor cells are relatively insensitive to chemotherapeutic drugs because the cell cycle is the mechanism by which cells are divided. Through inducing G2 and M phase arrest and inhibiting cell division, isoliquiritigenin has showed antitumor effect in a variety of tumors [143]. Using 3-MA to suppress autophagy induced by isoliquiritigenin enhances its antitumor activity in ES-2 cells [143]. In most cancer cell types, silibinin also causes cell cycle arrest, resulting in cell apoptosis [144]. Likewise, inhibition of autophagy increases silibinin-induced SW480 and SW620 cell death [145]. Another important anti-cell cycle chemotherapeutic agent, vincristine, induces apoptosis of gastric cancer cells, and siRNA knock-down Beclin1 or ATG5 sensitizes vincristine-resistance tumor cells [146]. Maybe the most interesting aspect of those drugs that inhibit cell cycle is how they initiate protective autophagy with one or more specific signal pathway(s) in various cell types. This will be more conducive to remove the drug resistance of tumors by autophagy.

Cetuximab, a therapeutic antibody that blocks the function of epidermal growth factor receptor, induced apoptosis and autophagy, and the knockdown of ATG7 or Beclin1 or treatment with CQ sensitized cancer cells to cetuximab-triggered apoptosis [147]. Mechanistically, cetuximab acted by downregulating Bcl-2 to promote the association between Beclin1 and VPS34. Human epidermal growth factor receptor 2 (HER2) is highly expressed in a variety of cells, such as breast carcinomas [148], colon cancer [149] and stomach adenocarcinoma [150]. Trastuzumab, as a humanized monoclonal antibody binding to domain IV of HER2, is approved by FDA for the treatment of HER2-positive breast and stomach adenocarcinoma/gastroesophageal junction adenocarcinoma cancer (https://www.cancer.gov/about-cancer/treatment/drugs/trastuzumab). The state of “autophagy addiction” also occurs in trastuzumab-resistant tumor cells. However, targeted genetic ablation of ATG5, ATG8 or ATG12 notably reduces the intrinsic refractory of trastuzumab [148]. Table 1 presents several examples of therapeutic anti-cancer antibodies, their targets and primary indications. These reports indicate that combined targeting autophagy can enhance the anti-cancer effect of the therapeutic antibodies.

**Table 1:** Summary of therapeutic antibodies combined with inhibition of autophagy

Therapeutic antibodies	Targets	Combination therapy	Indications	References
Rituximab	CD20	CQ	Non-Hodgkin lymphoma	[151]
Bevacizumab	VEGF	CQ	HCC	[152]
Cetuximab	EGFR	CQ	Vulvar squamous carcinoma	[147]
		Overexpressed Bcl-2	Colorectal adenocarcinoma	
		siRNA ATG7	Lung adenocarcinoma	
		siRNA Beclin1		
Gemtuzumab ozogamicin	CD33	PP242	Acute myeloid leukemia	[153]
		AZD2014		[154]
DN30	Met	Baf	Cardiomyoblasts	[155]
DO24				
Trastuzumab	HER2	CQ	Breast cancer	[148]
Milatuzumab	CD74	FTY720	Mantle cell lymphoma	[156]
Tositumomab	CD20	Overexpressed Bcl-2	B cell malignancies	[157]
		siRNA Beclin1		
		siRNA ATG 12		
CH12	EGFRvIII	siRNA ATG7	HCC	[158]
		siRNA Beclin1		
β2M mAb	β_2_-microglobulin	Bortezomib	Multiple myeloma	[159]
PD-1 mAb	PD-1	Pemetrexed + sildenafil	Non-small cell lung cancer	[160]
CTLA4 mAb	CTLA4			

As another important therapeutic strategy, miRNAs have been shown to regulate drug resistance in a wide variety of cancers. miR-23b-3p inhibited autophagy to sensitize vincristine-resistant gastric adenocarcinoma cell SGC7901 via decreasing the expression of ATG12 [161]. miRNA-29c inhibiting autophagy mediated by USP22 increased the gemcitabine-induced pancreatic cancer cell apoptosis [162]. Another recent study [163] showed that miR-409-3p ameliorated the sensitivity of ovarian cancer cells to cisplatin by interrupting the FIP200-mediated autophagy. However, all of them have been found to be downregulated in tumors, which may be one mechanism to result in drug resistance. Also, overcoming drug resistance by using miRNA to target autophagy may prove to be tempting and promising.

### Inducing autophagic cell death overcomes drug resistance

A large number of studies have shown that autophagy is necessary to effectively kill tumor cells in certain circumstances. Autophagy in this case is called autophagic cell death, which is regarded as cell death with autophagy rather than cell death by autophagy. Indeed, some drugs, such as rapamycin, bortezomib and butyrate to promote autophagosome formation [164, 165], and enforced expression of the autophagy-related genes, such as ATG3, ATG4 [166], ATG5 [164, 165], ATG9 [167] and Beclin1 [127], are demonstrated to induce autophagic cell death, and work in tandem with other chemotherapeutic drugs to conquer cancer in certain cell types.

As described early, mTOR is the central regulator of autophagy and a key target for autophagy regulation. The pharmacological inhibitor rapamycin enhanced isoliquiritigenin-induced autophagic and apoptotic cell death in cancer chemotherapy in adenoid cystic carcinoma cells [164]. The combination of everolimus, another mTOR inhibitor, and propachlor synergistically enhanced cell death by inducing autophagic cell death in prostate cancer cells [168]. Similarly, RAPA, a mTOR inhibitor to induce autophagy, increased cell death in temozolomide-treated U251 glioma cells [169]. However, inhibition of mTOR does not always induce autophagic death, but rather protective autophagy. For instance, given that mTOR inhibitor is a potent inducer of autophagy, HCQ relieved temsirolimus, a mTOR inhibitor, resistance and significantly enhanced antitumor activity with safety and acceptability in cancer patients with advanced solid tumors and melanoma [138]. Currently, the role of autophagy in cancer patients is being studied in multiple clinical trials with mTOR inhibitors (https://www.clinicaltrials.gov).

The success of bortezomib, a proteasome inhibitor, has been a standard-of-care therapy for malignancy multiple myeloma [165]. However, bortezomib is not responded in many patients and those patients commonly develop drug tolerance. A recent study shows that enforced expression of the ATG5 overcomes bortezomib-resistance hematologic malignancies [165]. A novel proteasome inhibitor marchantin M could directly trigger autophagic cell death via PI3K/AKT/mTOR pathway in prostate cancer cells [170]. In this regard, induction of sustained autophagy (autophagic cell death) seems to be required to compensate for severely damaged proteasome pathway. Thus, induced autophagic cell death maybe overcomes drug resistance to proteasome inhibitors.

Histone deacetylase (HDAC) is believed to regulate the epigenetic alterations which could restrain tumor suppressor genes and promote tumorigenesis. Consequently, inhibition of HDAC becomes an attractive anti-cancer therapeutic approach. HDAC inhibitor NCO-90/141 increased cell death via cytochrome *c*-mediated apoptosis and caspase-independent autophagic cell death in leukemic cell lines [171]. When apoptosis was inhibited, LAQ824 and panobinostat, two other HDAC inhibitors, triggered autophagic cell death in lymphoma cells and tumor xenograft model [172]. To overcome EGFR tyrosine kinase inhibitor resistance in T790M mutant lung cancer, the combination of suberoylanilide hydroxamic acid (SAHA) and either BIBW2992 or WZ4002, two tyrosine kinase inhibitors, induces autophagic cell death to enhance anti-tumor effect in PC-9G and H1975 cells and mouse xenografts [173]. Summing up the above, autophagic cell death is a way of compensation for cell death when the cells fail to undergo apoptosis.

There are also other specific chemical drugs that induce autophagic cell death and then relieve the resistance to therapeutic drugs. For instance, ABT-888, a PPRA inhibitor, increased the therapeutic efficacy of temozolomide-resistance glioma cells by inducing autophagic cell death [174]. A small-molecule inhibitor of Bcl-2, (-)-gossypol, triggered autophagic cell death and inhibited the growth of androgen-independent prostate cancer xenografts with high levels of Bcl-2 to resist apoptosis [175]. All of these indicate that induced autophagic cell death, as a strategy of synergistic therapy, can occur in multiple drug resistant tumors to circumvent resistance.

## Conclusions

Clinically, the therapeutic resistance resulting in the frequency of tumor progression is disappointing for all people including researchers and clinicians. It is urgent to clarify the mechanisms by which induce drug resistance of tumors. Autophagy inhibition has been presented to improve antitumor efficacy and overcome therapeutic resistance in various types of tumor cells both in vitro and in vivo, which makes it popular with many researchers. However, with the development of autophagy studies*,* autophagy shows a dual role of pro-death and pro-survival in tumor therapeutic resistance, which also makes it ambiguous in the eyes of researchers. In established cancers, autophagy increases metabolism to inactivate drug and support drug resistance. Indeed, certain anticancer drugs can also induce excessive or sustained autophagy, which leads to the death of tumor cells. Thus, the effect of autophagy induction on the therapeutic efficacy of therapeutic drugs, to a great extent, depends on the characteristics of treatment, type of tumor and stage of disease.

Systemic therapy with autophagy has been used as the clinical therapy for patients with drug resistance. The following questions still remain to be answered: (1) Do cytotoxic stimuli activate autophagy (related to cell survival) or autophagic cell death? (2) What is the mechanism of autophagy activated by cytotoxic stimuli? (3) How to achieve the optimal combinations by targeting which specific biological molecules in autophagy? (4) Does the combination elicit undesirable side effects in cancer patients after long-term autophagy inhibition or autophagy induction (autophagic cell death)? Due to the complex regulatory mechanisms and effects of autophagy, it provides a broad research space for us to elucidate the mysteries. However, it also requires us to provide valuable information on how autophagy is manipulated during tumor drug resistance in future research.

## Data Availability

Not applicable.

## Abbreviations

3-MA: 3-Methyladenine
ALDH1A3: Aldehyde dehydrogenase 1A3
AMBRA-1: Autophagy/Beclin1 regulator-1
AMPK: Adenosine monophosphate kinase
ASK1: Apoptosis signal-regulating kinase 1
ATF4: Activating transcription factor 4
ATM: Ataxia telangiectasia mutated
BafA: Bafilomycin A1
BH3: Bcl-2-homology-3 domain
CaMKKβ: Calmodulin-dependent protein kinase kinase-β
CASC9: Cancer susceptibility candidate 9
CCAT1: Colon cancer-associated transcript-1
CCD: Coiled-coiled domain
C/EBP-β: CCAAT/enhancer-binding proteinβ
CHOP: C/EBP homologous protein
CLRN1-AS1: Clarin 1 antisense RNA 1
CML: Chronic myelogenous leukemia
CQ: Chloroquine
CTSD: Cathepsin D
DAPK1: Death-associated protein kinase 1
DDIT3/4: DNA damage-inducible transcript 3/4
DRAM: Damage-regulated autophagy modulator
DUSP4/5: Dual-specificity phosphatase 4/5
eIF2α: Eukaryotic initiation factor 2α
EPI: Epirubicin
ER: Endoplasmic reticulum
ERK1/2: Extracellular signal-regulated kinase1/2
FAK: Focal adhesion kinase
FIP200: Focal adhesion kinase (FAK) family interacting protein 200
FOXO: Forkhead box O
GAS5: Growth arrest‑specific 5
HCQ: Hydroxychloroquine
HDAC: Histone deacetylase
HER2: Human epidermal growth factor receptor 2
HIF: Hypoxia-inducible factor
HMGB1: High-mobility group B1
HSC70: Heat shock cognate 70
INSR: Insulin receptor
JNK: C-Jun N-terminal protein kinase
LAPTM4A: Lysosomal-associated transmembrane protein 4A
LC3-I/II: Microtubule-associated protein I/II light chain 3
LKB1: Liver kinase B1
lncRNAs: Long noncoding RNAs
MAPK: Mitogen-activated protein kinase
MDR: Multidrug resistance
MEFs: Mouse embryonic fibroblasts
MEG3: Maternally expressed gene 3
miR: MicroRNA (miRNA)
MMP: Mitochondrial membrane permeabilization
MRP1: Multidrug resistance-associated protein 1
mTOR: Mammalian target of rapamycin
NEAT1: Nuclear-enriched abundant transcript 1
PA: Palmitic acid
PARP: Poly(ADP-ribose) polymerase
P-gp: P-glycoprotein
PKR: Protein kinase R-like endoplasmic reticulum kinase
PtdIns(3)P: Phosphotidylinositol triphosphate
PTEN: Phosphatase and tensin homolog
PVT1: Plasmacytoma variant translocation1
Rheb: Ras homolog enriched in brain
RNS: Reactive nitrogen species
ROS: Reactive oxygen species
SAHA: Suberoylanilide hydroxamic acid
siRNA: Small interfering RNA
SQSTM1: Sequestosome1
TPP1: Tripeptidyl peptidase 1
TRAF2: TNF receptor-associated factor 2
TRAIL: TNF-related apoptosis-inducing ligand
TSC: Tuberous sclerosis complex
ULK1/2: Unc-51-like kinase-1/2
UPR: Unfolded protein response
UTR: Untranslated regions
UVRAG: UV radiation resistance-associated gene protein
VPS34: Vacuor protein sorting-34
XBP1: X box-binding protein 1
